# DSAC-ICM: A Distributional Reinforcement Learning Framework for Path Planning in 3D Uneven Terrains

**DOI:** 10.3390/s26030853

**Published:** 2026-01-28

**Authors:** Yixin Zhou, Fan Liu, Zhixiao Liu, Xianghan Ji, Guangqiang Yin

**Affiliations:** 1 School of Automation Engineering, University of Electronic Science and Technology of China, Chengdu 611731, China; zyx_uestc2025@163.com; 2 School of Information and Software Engineering, University of Electronic Science and Technology of China, Chengdu 611731, China; 3 Glasgow College, University of Electronic Science and Technology of China, Chengdu 611731, China; 4 School of Computer Science and Engineering, University of Electronic Science and Technology of China, Chengdu 611731, China

**Keywords:** distributional reinforcement learning, soft actor–critic, intrinsic curiosity module, global path planning, 3D uneven terrain

## Abstract

Ground autonomous mobile robots are increasingly critical for reconnaissance, patrol, and resupply tasks in public safety and national defense scenarios, where global path planning in 3D uneven terrains remains a major challenge. Traditional planners struggle with high dimensionality, while Deep Reinforcement Learning (DRL) is hindered by two key issues: (1) systematic overestimation of action values (Q-values) due to function approximation error, which leads to suboptimal policies and training instability; and (2) inefficient exploration under sparse reward signals. To address these limitations, we propose DSAC-ICM: a Distributional Soft Actor–Critic framework integrated with an Intrinsic Curiosity Module (ICM). Our method fundamentally shifts the learning paradigm from estimating scalar Q-values to learning the full probability distribution of state-action returns, which inherently mitigates value overestimation. We further integrate the ICM to generate dense intrinsic rewards, guiding the agent toward novel and unvisited states to tackle the exploration challenge. Comprehensive experiments conducted in a suite of realistic 3D uneven-terrain environments demonstrate that DSAC-ICM successfully enables the agent to learn effective navigation capabilities. Crucially, it achieves a superior trade-off between path quality and computational cost when compared to traditional path planning algorithms. Furthermore, DSAC-ICM significantly outperforms other RL baselines in terms of convergence speed and return.

## 1. Introduction

Autonomous navigation in complex natural environments has become one of the key challenges for mobile robots [1,2]. In applications such as geological exploration, planetary rovers, and disaster rescue, robots must traverse three-dimensional uneven terrains, where elevation variations, irregular slopes, and uncertain traversability significantly complicate motion planning [3]. Path planning, which aims to generate an optimal feasible trajectory from a start to a goal position while minimizing cost functions such as distance, energy consumption, or time, plays a central role in achieving autonomous navigation. However, global path planning is a well-known NP-Hard problem, and its computational complexity grows rapidly with dimensionality [4].

Traditional path planning algorithms—such as Dijkstra [5], A* [6], and Rapidly-exploring Random Tree (RRT) [7]—have been extensively studied and widely applied in structured environments. These methods rely on search strategies. While they can efficiently find collision-free paths in low-dimensional static environments, their performance deteriorates on uneven terrains, where the cost of traversing each region is strongly influenced by terrain elevation and slope. Moreover, when the environment changes, these algorithms must reconstruct the search graph or tree, leading to poor adaptability and limited scalability [3]. Beyond classical single-agent path planning, several studies have explored navigation problems under more complex constraints. For instance, task assignment for multiple vehicles with partially unreachable targets focuses on coordination and feasibility at the decision-making level rather than low-level motion planning [8]. Path planning in uncertain environments with moving obstacles has also been investigated using warm-start cross-entropy methods to improve robustness under dynamic conditions [9]. In addition, multi-objective optimization-based approaches, such as the 3D-M method, jointly consider distance and energy consumption for path planning on uneven 3D terrains [10]. While effective in modeling terrain-induced costs, these methods typically rely on predefined cost formulations and optimization heuristics, and do not explicitly address continuous-control learning in complex 3D environments. Moreover, it is worth noting that most global path planning methods, including both classical optimization-based approaches and learning-based planners, are commonly formulated under static and idealized environment assumptions. Such formulations focus on long-horizon route optimization based on prior terrain information, while deferring real-time adaptability to lower-level planning or control modules.

From a computational perspective, reinforcement learning-based planners and traditional search-based planners also exhibit fundamentally different characteristics. For small-scale maps or single-shot planning tasks, classical algorithms such as Dijkstra or A* are often more computationally efficient, as they do not require a training phase. In contrast, RL-based methods incur higher computational cost during training, but this cost is amortized over repeated deployments. Once trained, policy inference requires only a forward pass through a neural network, resulting in near-constant planning time and enabling efficient reuse across different start–goal configurations.

In contrast, reinforcement learning (RL) offers a data-driven approach for learning navigation policies [11]. Agents improve through trial-and-error interactions with the environment [12]. By updating policies and value functions based on observed rewards, RL enables adaptation to dynamic or partially known environments. RL algorithms can be classified as on-policy or off-policy.

Off-policy algorithms such as DDPG [13], TD3 [14], and SAC [15] are sample-efficient and suitable for high-dimensional continuous control. However, they often suffer from Q-value overestimation [16,17], which in uneven terrain navigation can lead to unsafe or infeasible paths that appear optimal in the learned value function. Additionally, sparse and delayed goal-reaching rewards limit exploration, causing the agent to get stuck in local minima and fail to find traversable routes [18]. Together, **overestimation** and **sparse rewards** make stable and efficient global path planning in 3D uneven terrains particularly challenging.

To address these challenges, we propose DSAC-ICM, a reinforcement learning framework for 3D path planning in uneven terrains. Our approach integrates the Distributional Soft Actor–Critic (DSAC) algorithm [19,20] with an Intrinsic Curiosity Module (ICM) [21] to jointly enhance value estimation stability and exploration capability. Specifically, DSAC models the full return distribution rather than its expectation, which mitigates Q-value overestimation and improves robustness against high-variance terrain dynamics. Meanwhile, ICM provides an intrinsic reward signal that promotes efficient exploration in sparse-reward environments, guiding the agent toward informative states and reducing the likelihood of premature convergence. The combination enables the agent to learn safer, smoother, and more energy-efficient global paths that better adapt to complex topographies. The main contributions are summarized as follows:

- We propose a reinforcement learning-based global path planning framework for 3D uneven terrains, which explicitly considers robot motion constraints, supports continuous action spaces, and optimizes for traversed distance over uneven terrain.
- We design a DSAC-ICM algorithm, which combines distributional value learning with curiosity-driven intrinsic motivation to jointly address Q-value overestimation and sparse-reward exploration.
- We conduct comprehensive experiments on DEM-based 3D terrain datasets, showing that DSAC-ICM performs well in path optimality, learning stability, and exploration efficiency.

The remainder of this paper is organized as follows. We first formalize the 3D path planning problem and present the Markov decision process formulation in Section 3. Section 4 describes the proposed DSAC-ICM framework, including its distributional critic and curiosity-driven exploration strategy. Experimental results and analysis are presented in Section 5, and conclusions and future directions are discussed in Section 6.

## 2. Related Work

Over the past decades, a wide range of algorithms have been proposed for global path planning. Classical methods include graph-based algorithms such as Dijkstra [5] and A* [6], which guarantee optimal solutions but suffer from high computational costs on large or complex terrains, and sampling-based methods like RRT [7] and RRT* [22], which scale well to high-dimensional spaces but require frequent replanning in dynamic environments. Bio-inspired algorithms and heuristic search approaches have also been explored to approximate optimal paths or incorporate multi-objective criteria such as energy consumption, slope constraints, or scenic routes [3]. While these methods are effective in structured or flat environments, they struggle with uneven 3D terrains where path cost depends on both horizontal displacement and elevation changes, and where safe traversal requires consideration of slope feasibility and energy consumption. Consequently, traditional planners often fail to simultaneously satisfy physical constraints, minimize traversal cost, and maintain computational efficiency in complex 3D terrains.

Among learning-based methods, reinforcement learning (RL) has emerged as a promising alternative for path planning [23], enabling agents to discover feasible and efficient trajectories through interaction with the environment. Recent deep RL (DRL) applications include UAV-based weed localization using DQN [24], urban IoT data collection via convolutional networks [25], hierarchical DRL for indoor navigation with LiDAR-based complexity metrics [26], and attention-guided TERP for rugged terrains [27]. Off-policy algorithms, such as DQN [28], DDPG [13], TD3 [14], and SAC [15], are particularly suitable for continuous control due to their sample efficiency and stability, but often suffer from Q-value overestimation and unstable learning in high-variance environments such as 3D uneven terrains [16,17]. To mitigate overestimation, methods such as Double Q-learning [29] and Double DQN [30] decouple action selection from evaluation in discrete spaces. Continuous control extensions, such as Double DDPG [14], reduce but do not fully eliminate overestimation, while Clipped Double Q-learning, used in TD3 and SAC, partially alleviates overestimation by taking the minimum of two Q-estimates but may introduce underestimation bias. Distributional RL methods, including Distributional SAC (DSAC) [19,20], address these issues by modeling the full return distribution, capturing uncertainty across returns and improving value estimation stability in complex or high-variance environments. In addition to value decomposition and distributional modeling, reward shaping-based approaches have also been proposed to mitigate value estimation bias in off-policy reinforcement learning. Munchausen Reinforcement Learning (M-RL) incorporates a log-policy term directly into the reward function, inspired by entropy-regularized RL, thereby reshaping the Bellman target and reducing overestimation effects [31]. By augmenting the reward with a scaled log-policy term, M-RL encourages more conservative value updates and has been shown to improve learning stability in several continuous control tasks. However, as a reward-level modification, its effectiveness may still be limited in environments characterized by highly sparse rewards and strong return variance induced by complex terrain dynamics.

Another critical challenge in path planning is sparse reward signals, where meaningful feedback is only obtained when the agent reaches or approaches the goal. Curiosity-driven exploration mechanisms, such as the Intrinsic Curiosity Module (ICM) [21], provide intrinsic reward signals based on state prediction errors, guiding the agent to explore informative states and improving training efficiency in sparse-reward scenarios. To further enhance exploration stability in continuous action spaces, ICM has been integrated with TD3, introducing a randomness-enhanced module to encourage exploration of unknown regions and reduce local optima [32]. Multi-agent sparse-reward scenarios have been addressed with the I-Go-Explore framework, which combines ICM with Go-Explore to leverage historical exploration experience for targeted state visitation and improved sample efficiency [33].

## 3. Problem Formulation

In this paper, we consider the problem of global path planning for ground mobile robots operating in uneven 3D terrains, where the objective is to generate a feasible path from a start position to a target position while respecting terrain-dependent physical constraints. Compared with flat terrain, path planning in uneven environments is more challenging because the robot must additionally account for slope variations and its maximum admissible slope constraint.

The uneven terrain is modeled as a 2D grid map consisting of *N* nodes. Each node *i* is associated with an elevation value H(i) derived from a Digital Elevation Map (DEM). Let the start and goal positions be denoted as $s_{0}$ and $s_{g}$, respectively.

The robot moves with a fixed step length *l*, and at each decision step selects a moving direction from a continuous action space. Therefore, the action space is defined as:

(1) $$A=\{a\in R^{2}|\;∥a∥=l\},$$

i.e., the action corresponds to selecting a continuous movement direction, and the robot moves to the next state by

(2) $$s_{t+1}=s_{t}+a.$$

During the planning process, robot motion must satisfy the slope constraint physical constraint. The slope angle between two adjacent nodes *i* and *j* must not exceed the robot’s maximum admissible slope $\theta_{\max}$. The slope angle is computed as:

(3) $$\theta \left(i,j\right)=\arctan\left(\frac{H(j)-H(i)}{d(i,j)}\right),$$

where d(i,j) denotes the Euclidean distance between nodes *i* and *j* in the horizontal plane.

The goal of path planning is to generate a sequence of states and actions that starts from $s_{0}$, ends at $s_{g}$, satisfies the slope constraint, and minimizes the total path cost. The path cost is defined as the sum of the movement costs along the path:

(4) $$J\left(\tau \right)=\sum_{t=0}^{T-1}c\left(s_{t},s_{t+1}\right),$$

where the unit movement cost is given by

(5) $$c\left(s_{t},s_{t+1}\right)=\sqrt{d{\left(s_{t},s_{t+1}\right)}^{2}+{\left(H\left(s_{t+1}\right)-H\left(s_{t}\right)\right)}^{2}}.$$

We formulate the path planning problem as a Markov Decision Process (MDP), defined by a tuple 〈S,A,R,γ〉:

- **State space **S**.** Includes the elevation matrix *H*, the robot’s current position, and the target position.
- **Action space **A**.** A continuous action space representing the movement direction with fixed step length.
- **Reward function **R**.** The immediate reward r=R(s,a) is determined by the current state and action.
- **Discount factor **γ**.** A scalar γ∈(0,1] used to compute the cumulative return.

To clearly define the scope of this study, we make several assumptions: the robot has complete prior knowledge of the terrain elevation data and full awareness of its own state, including position and actions. The environment is assumed to be static during the global planning process, which allows the planner to focus on long-horizon route optimization based on known terrain elevation data. Handling dynamic obstacles and real-time disturbances is beyond the scope of this work and is typically addressed by a local planner or reactive control layer.

Ideally, the path planning algorithm aims to find a feasible path $\tau^{*}$ that minimizes the cost:

(6) $$\tau^{*}=\arg\min_{\tau }J\left(\tau \right),$$

which is equivalent to maximizing the cumulative reward in the MDP formulation. However, global path planning is an NP-hard problem, making it computationally infeasible to guarantee global optimality in polynomial time. Therefore, practical approaches aim to compute, within reasonable time, a path that satisfies physical constraints and achieves as low a cost as possible.

In this work, we focus on improving planning efficiency and path optimality for robots navigating uneven terrains, while ensuring compliance with slope constraints and maintaining robustness and computational efficiency.

## 4. Method

### 4.1. Method Overview

We propose DSAC-ICM, a novel reinforcement learning framework that integrates Distributional Soft Actor–Critic (DSAC) with the Intrinsic Curiosity Module (ICM) to achieve robust and efficient global path planning in complex 3D uneven terrains. Our framework specifically addresses the dual challenges of Q-value overestimation prevalent in standard off-policy algorithms and sparse exploration inherent in goal-reaching tasks. The overall architecture is based on the actor–critic paradigm, utilizing a continuous action space for flexible movement direction selection.

### 4.2. Overestimation in 3D Terrain Path Planning

Off-policy actor–critic algorithms such as DDPG, TD3, and SAC approximate value functions using neural networks and update them via Bellman backups. Due to approximation noise and the implicit max operator in policy improvement, these methods commonly suffer from Q-value overestimation. In SAC, for example, the one-step target is

(7) $$y=r+\gamma \left(Q_{\bar{\theta }}\left(s^{\prime },a^{\prime }\right)-\alpha \log\pi \left(a^{\prime }|s^{\prime }\right)\right),$$

where noise in $Q_{\bar{\theta }}$ is directly propagated into optimistic targets, causing error accumulation and unstable learning.

This issue becomes more severe in 3D uneven terrain path planning. First, transition dynamics exhibit high variance because movement cost depends on both horizontal displacement and elevation change; slight action perturbations can cause large height variations and reward noise, leading the critic to favor steep “shortcut” directions. Second, the slope constraint $\theta \left(i,j\right)\leq \theta_{\max}$ creates a hard feasibility boundary. Overestimated critics often assign high value to actions near or beyond this boundary, promoting transitions that are physically infeasible. Third, sparse goal-reaching rewards make early training dominated by bootstrapping, amplifying the accumulation of optimistic bias. As a result, SAC-based planners may prefer steep or unsafe trajectories that appear advantageous in the value approximation but violate terrain constraints or lead to unstable behavior.

### 4.3. Distributional Soft Actor–Critic

**Figure 1:**
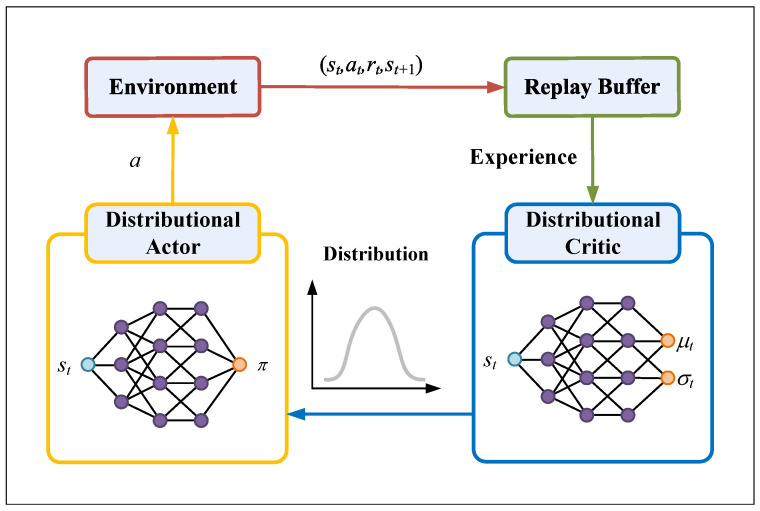
Distributional Soft Actor–Critic.

Conventional SAC estimates the expected return using a scalar critic, which is prone to overestimation in noisy or high-variance environments such as 3D uneven terrains. To address this limitation, Distributional Soft Actor–Critic (DSAC) extends SAC by modeling the full distribution of soft state-action returns $Z^{\pi }\left(s,a\right)$ instead of a single mean. This enables the critic to capture uncertainty in value estimates, reducing overestimation and promoting safer policy learning in complex terrains. Figure 1 illustrates the overall DSAC architecture.

DSAC treats the soft return as a random variable:

(8) $$Z^{\pi }\left(s,a\right)\approx T^{\pi }Z^{\pi }\left(s,a\right),$$

where the distributional Bellman operator is defined as

(9) $$T^{\pi }Z^{\pi }\left(s,a\right)\overset{D}{=}r+\gamma \left(Z^{\pi }\left(s^{\prime },a^{\prime }\right)-\alpha \log\pi \left(a^{\prime }|s^{\prime }\right)\right),$$

with $s^{\prime }∼p\left(\cdot |s,a\right)$, $a^{\prime }∼\pi \left(\cdot |s^{\prime }\right)$, and r∼R(·|s,a). A parametric approximation $Z_{\theta }\left(s,a\right)$ is maintained and updated by minimizing a divergence d(·,·), specifically the Kullback–Leibler (KL) divergence, between the target distribution and the predicted distribution:

(10) $$\theta \leftarrow \arg\min_{\theta }E_{(s,a)∼D}\left[d\left(T^{\pi }Z_{\bar{\theta }}\left(s,a\right),Z_{\theta }\left(s,a\right)\right)\right],$$

where $Z_{\bar{\theta }}$ is the target critic.

DSAC typically maintains two critics and one actor, similar to SAC. The critics output a parameterized approximation of Z(s,a), and a target network stabilizes learning. The actor is trained to maximize the expected return under the distributional critic:

(11) $$J_{\pi }=E_{s∼D,a\;∼\pi }\left[\alpha \log\pi \left(a|s\right)-E[Z_{\theta }\left(s,a\right)]\right].$$

By modeling the full return distribution, DSAC provides several key advantages for 3D uneven terrain navigation. First, it reduces overestimation by capturing uncertainty and preventing overly optimistic Q spikes near steep slopes or unstable transitions. Second, distributional targets smooth the high variance caused by irregular terrain dynamics, leading to more stable learning. Third, actions with risky or high-variance returns are naturally penalized, enabling safer policy learning that respects terrain constraints and robot stability. These features make DSAC more robust and reliable than conventional SAC in complex navigation tasks with nonlinear constraints and sparse rewards.

### 4.4. Intrinsic Motivation for Exploration

**Figure 2:**
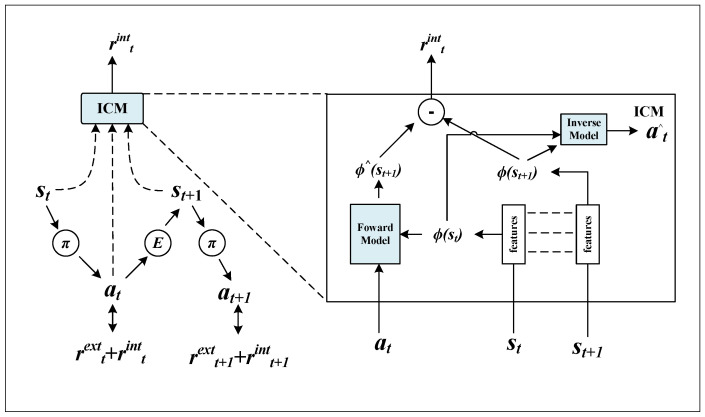
Intrinsic Curiosity Module.

In 3D uneven terrain navigation, the reward signal is typically sparse, as significant feedback is only obtained when the robot approaches the goal. This sparsity limits the agent’s ability to explore effectively, often causing the policy to converge prematurely to suboptimal routes. To address this challenge, we incorporate an Intrinsic Curiosity Module (ICM) that generates an auxiliary reward based on the agent’s prediction error of environment dynamics (Figure 2).

Formally, ICM consists of a forward model $f_{F}$ and an inverse model $f_{I}$. Given a transition $\left(s_{t},a_{t},s_{t+1}\right)$, the inverse model predicts the action ${\hat{a}}_{t}=f_{I}\left(s_{t},s_{t+1}\right)$, while the forward model predicts the next state embedding ${\hat{\phi }}_{t+1}=f_{F}\left(\phi \left(s_{t}\right),a_{t}\right)$, where ϕ(s) is a learned feature representation of the state. The intrinsic reward is defined as the prediction error of the forward model:

(12) $$r_{t}^{\mathrm{int}}=\eta \;{∥{\hat{\phi }}_{t+1}-\phi \left(s_{t+1}\right)∥}^{2},$$

where η is a scaling factor. This reward encourages the agent to visit states that are novel or hard to predict, effectively guiding exploration in sparse-reward environments.

The total reward used for policy learning combines the environment reward and intrinsic reward:

(13) $$r_{t}^{\mathrm{total}}=r_{t}^{\mathrm{ext}}+\lambda \;r_{t}^{\mathrm{int}}.$$

where λ controls the contribution of curiosity-driven reward.

By leveraging intrinsic motivation through ICM, the agent can efficiently explore complex 3D terrains, discover feasible paths, and overcome the limitations imposed by sparse goal-reaching rewards.

### 4.5. Reward Function

The design of the reward function is critical for guiding the agent to navigate effectively and safely in 3D uneven terrains. Our reward structure consists of several components reflecting environmental constraints and providing informative feedback at each step.

A positive reward, $r_{\mathrm{goal}}$, is granted when the agent reaches the goal. If a proposed action leads the agent outside the valid map boundaries, the action is aborted and a collision penalty, $r_{\mathrm{coll}}$, is applied. Slope constraints are enforced by computing the slope along the proposed movement. If the slope exceeds the robot’s maximum traversable angle, $\theta_{\max}$, the move is allowed but a negative slope penalty, $r_{\mathrm{slope}}$, is incurred, guiding the agent to avoid overly steep terrain.

To provide denser feedback, a progress-based reward, $r_{\mathrm{progress}}$, is given according to the reduction in horizontal distance to the goal:

(14) $$r_{\mathrm{progress}}=w_{\mathrm{progress}}\cdot \left({\mathrm{dist}}_{\mathrm{horiz},t-1}-{\mathrm{dist}}_{\mathrm{horiz},t}\right),$$

where the horizontal distance is computed as

(15) $${\mathrm{dist}}_{\mathrm{horiz},t}=\sqrt{{\left(x_{t}-x_{\mathrm{goal}}\right)}^{2}+{\left(y_{t}-y_{\mathrm{goal}}\right)}^{2}},$$

and $w_{\mathrm{progress}}$ is a positive scaling factor. Additionally, a path efficiency reward penalizes the 3D Euclidean distance traveled in each step:

(16) $$r_{\mathrm{path}}=-w_{\mathrm{path}}\cdot d_{3D\_\mathrm{step}},$$

where the 3D step length is

(17) $$d_{3D\_\mathrm{step}}=\sqrt{{\left(x_{t}-x_{t-1}\right)}^{2}+{\left(y_{t}-y_{t-1}\right)}^{2}+{\left(z_{t}-z_{t-1}\right)}^{2}},$$

discouraging unnecessarily long or circuitous paths while respecting vertical variations.

A small negative step penalty, $r_{\mathrm{step}}$, is applied at each step to encourage shorter trajectories.

The total external reward at each time step is the sum of all components:

(18) $$r_{t}=r_{\mathrm{goal}}+r_{\mathrm{coll}}+r_{\mathrm{slope}}+r_{\mathrm{progress}}+r_{\mathrm{path}}+r_{\mathrm{step}}.$$

This reward design ensures the learned policy is goal-directed, safe, and physically plausible, effectively promoting efficient navigation across challenging 3D uneven terrains.

## 5. Experimental

### 5.1. Experimental Setup

#### 5.1.1. Datasets

**Figure 3:**
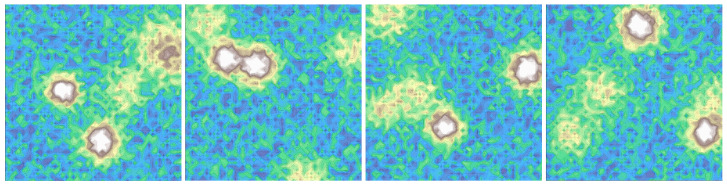
Four sample from the terrain datasets.

The experiments are conducted on a custom set of 20 synthetic 3D terrain maps, each with a size of 100×100 grid cells. The terrains are designed to simulate complex uneven environments with varying slopes and elevations. Each map contains a mixture of topographic features, including randomly generated high-elevation regions resembling mountains that are completely impassable, moderate hilly areas that can be navigated with careful path planning, and relatively flat regions with small random perturbations that introduce subtle irregularities. This diverse combination of terrain features ensures that the agent encounters a wide range of navigation challenges, from entirely blocked paths to subtle elevation changes requiring precise movement. Figure 3 illustrates four samples from the dataset. Although the terrains are synthetically generated, they are designed to cover a wide range of elevation patterns and slope distributions, serving as controlled testbeds for evaluating global planning performance under uneven terrain conditions.

#### 5.1.2. Evaluation Metrics

To comprehensively evaluate the performance of DSAC-ICM, we employ multiple metrics:

- **Average Return (AR):** The mean cumulative reward per episode, measured over training iterations. This reflects the learning progress and convergence of the policy.
- **Path Cost (PC):** The total 3D distance traversed by the agent along the planned trajectory. Unlike 2D horizontal distance, this accounts for elevation changes and better reflects energy expenditure and traversal effort.
- **Path Cost Ratio (PCR):** The ratio between the agent’s path cost and the optimal path cost computed by Dijkstra’s algorithm in the same 3D terrain. Values closer to 1 indicate near-optimal paths.
- **Planning Time Ratio (PTR):** The ratio of the agent’s planning time to Dijkstra’s planning time. Lower values correspond to higher efficiency.
- **Cost-Time Tradeoff (CTT):** A novel metric introduced in this work that combines path cost and planning time to provide a single measure of efficiency:
  (19) $$CTT=PCR\cdot \log\left(1+PTR\right).$$

### 5.2. Experimental Settings

This section summarizes the hyperparameters, software setup, and implementation details used for training the DSAC-ICM agent and all baseline algorithms. All methods are implemented in Python using PyTorch, and experiments are conducted under the same environment for fair comparison. The key training, network, and exploration parameters are listed in Table 1. These include the actor–critic learning rates, ICM configuration, replay buffer size, discount factors, update frequencies, and main architectural choices.

The ICM forward loss weight β determines the contribution of the forward model prediction error in the total ICM loss. The intrinsic reward is defined as $r_{t}^{\mathrm{int}}=\eta {∥{\hat{\phi }}_{t+1}-\phi \left(s_{t+1}\right)∥}^{2}$, where η scales the prediction error. The intrinsic reward weight λ balances exploration and task-driven optimization in $r_{t}^{\mathrm{total}}=r_{t}^{\mathrm{ext}}+\lambda r_{t}^{\mathrm{int}}$. These three parameters are critical for guiding exploration in sparse-reward environments.

All experiments run on a workstation equipped with an Intel(R) Xeon(R) Gold 6226R CPU @ 2.90 GHz, a single NVIDIA Tesla T4 GPU with 16 GB memory, and Ubuntu 22.04.4 LTS. The implementation uses Python 3.12.0 and PyTorch 2.5.1 with CUDA 12.4 support. To ensure reproducibility, all experiments are conducted under three different random seeds, and the results presented in the figures and tables represent the mean standard deviation across these runs.

### 5.3. Experimental Results

**Figure 4:**
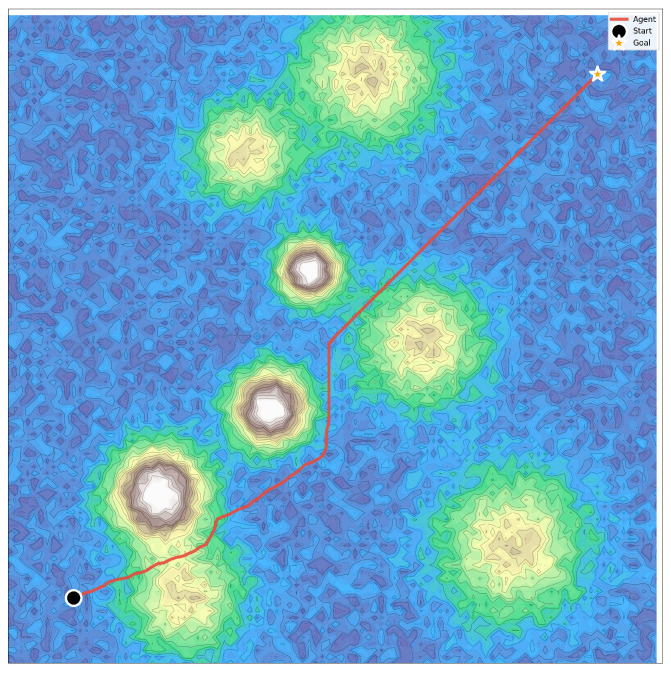
Path planning result.

This section presents a comprehensive evaluation of the proposed DSAC-ICM framework for 3D uneven terrain path planning. The experiments are conducted on multiple terrain datasets. Figure 4 shows a representative 2D projection of one uneven terrain instance. The black dot denotes the start point, and the orange star marks the target. The red line indicates the trajectory generated by our DSAC-ICM planner. As shown in the figure, the agent successfully identifies a feasible and collision-free path that reaches the goal while avoiding steep mountainous regions. This behavior reflects the agent’s ability to respect the maximum traversable slope constraint and optimize long-horizon navigation under complex elevation variations.

Table 2 reports the quantitative comparison among classical algorithms (Dijkstra, A*, RRT) and our method. Four metrics are considered: Path Cost Ratio (PCR), Planning Time Ratio (PTR), and the proposed Cost-Time Tradeoff (CTT). Dijkstra is used as the reference optimal planner in terms of path cost. A* achieves similar optimality (PCR = 1.000) and improves planning time efficiency (PTR = 0.196), but its CTT remains limited due to its high dependence on heuristic expansions. RRT exhibits significantly higher path cost (PCR = 1.159) and considerably slower planning time (PTR = 0.870), resulting in a large CTT value. The 3D-M method achieves relatively fast planning efficiency (PTR = 0.098), but this advantage is obtained at the expense of path quality, resulting in a higher path cost ratio (PCR = 1.242) and a limited cost-time tradeoff (CTT = 0.106).

**Figure 5:**
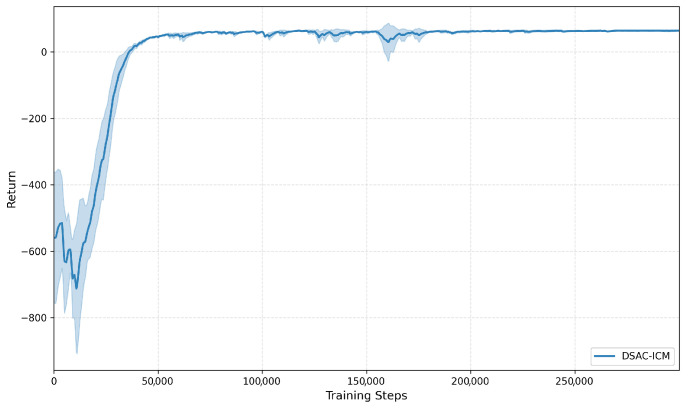
Training return curve of DSAC-ICM. The horizontal axis represents training steps, and the vertical axis denotes the training return.

In contrast, DSAC-ICM achieves a favorable balance between path optimality and computational efficiency. Although the average PCR (1.137) is slightly higher than that of A*, the planning time requirement is drastically reduced (PTR = 0.056). The combined effect leads to the lowest CTT score (0.055), indicating that DSAC-ICM offers the most efficient overall tradeoff between path quality and computation. The stable training process of its underlying framework (DSAC) further supports this superior performance, as shown in Figure 5: in the early training stage (steps < 50,000), the return fluctuates and stays at a low level, reflecting the unstable strategy during initial exploration; when steps reach 50,000, the return rises rapidly and converges to a stable range close to 0, which means the model’s strategy gradually optimizes and matures, laying a solid foundation for subsequent task performance. This superior performance can be attributed to the intrinsic curiosity module, which enhances exploration in unfamiliar topographic regions, and the distributional critic, which improves value estimation under elevation-driven uncertainty.

Overall, these results demonstrate that DSAC-ICM not only generates feasible and smooth trajectories on highly uneven 3D terrains but also achieves competitive optimality with significantly lower planning cost. This makes it suitable for real-time autonomous navigation in outdoor environments with complex elevation profiles.

Following the above results, we further compare the learning performance of DSAC-ICM with several mainstream deep reinforcement learning algorithms, including A2C [34], PPO [35], SAC [15], and M-RL [31]. Since the path planning task is formulated with a continuous action space, we implement Munchausen Reinforcement Learning using its SAC-based variant, ensuring a fair comparison under the same continuous-control setting. To provide a direct comparison of policy learning efficiency, we plot the learning curves with the number of environment interaction steps on the horizontal axis (*steps*) and the episodic return on the vertical axis (*return*). This visualization highlights how quickly each algorithm discovers effective policies and how stable their final performance is.

**Figure 6:**
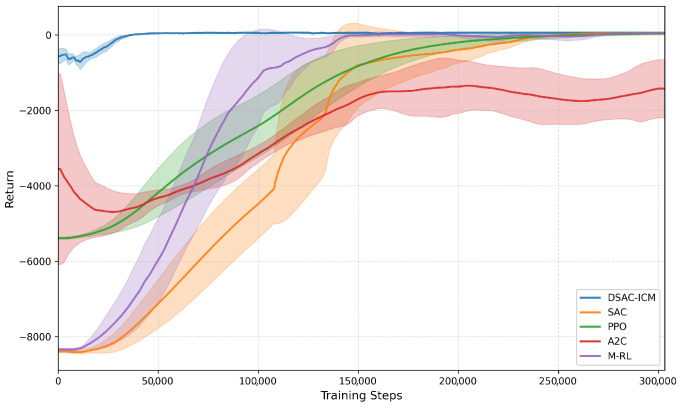
Training return curves of different deep reinforcement learning algorithms. The horizontal axis denotes the number of environment interaction steps, and the vertical axis represents the episodic return. The curves correspond to DSAC-ICM (blue), A2C (orange), PPO (green), SAC (red), and M-RL (purple), respectively.

As illustrated in Figure 6, all learning-based methods except A2C are able to achieve positive returns during training, indicating successful policy learning in the 3D uneven-terrain environment. Among them, DSAC-ICM (blue) converges faster and more consistently to the highest return regime among all compared methods, exhibiting both strong final performance and stable learning behavior. SAC (red) and M-RL (purple) also achieve positive returns, but exhibit either slower convergence or higher performance variance compared to DSAC-ICM. PPO (green) improves steadily but plateaus at a substantially lower return level, while A2C (orange) fails to obtain meaningful rewards within the same training budget.

Specifically, DSAC-ICM exhibits the fastest convergence and the best final performance: after a short exploration phase (steps < 50,000), it rises rapidly and stabilizes at the highest return plateau. SAC also converges to a positive-return level, but its convergence is slower, and its asymptotic return is lower than that of DSAC-ICM. Notably, M-RL shows slightly improved early-stage learning dynamics compared to SAC, exhibiting faster initial improvement, which can be attributed to Munchausen reward shaping that mitigates overly optimistic value updates under sparse and noisy terrain rewards.

Overall, these results suggest that the combination of distributional value estimation and intrinsic curiosity enables DSAC-ICM to explore more efficiently and learn more reliably under elevation-induced reward uncertainty.

In addition to the learning curves shown in Figure 6, the quantitative comparison of final episodic returns across reinforcement learning algorithms is summarized in Table 3. Each value represents the mean standard deviation computed over three independent runs with different random seeds.

To further investigate the contribution of the Intrinsic Curiosity Module (ICM), we conduct an ablation study by training DSAC with and without ICM. Similar to the previous setup, we plot the learning curves using *steps* (horizontal axis) and *return* (vertical axis) for comparison.

**Figure 7:**
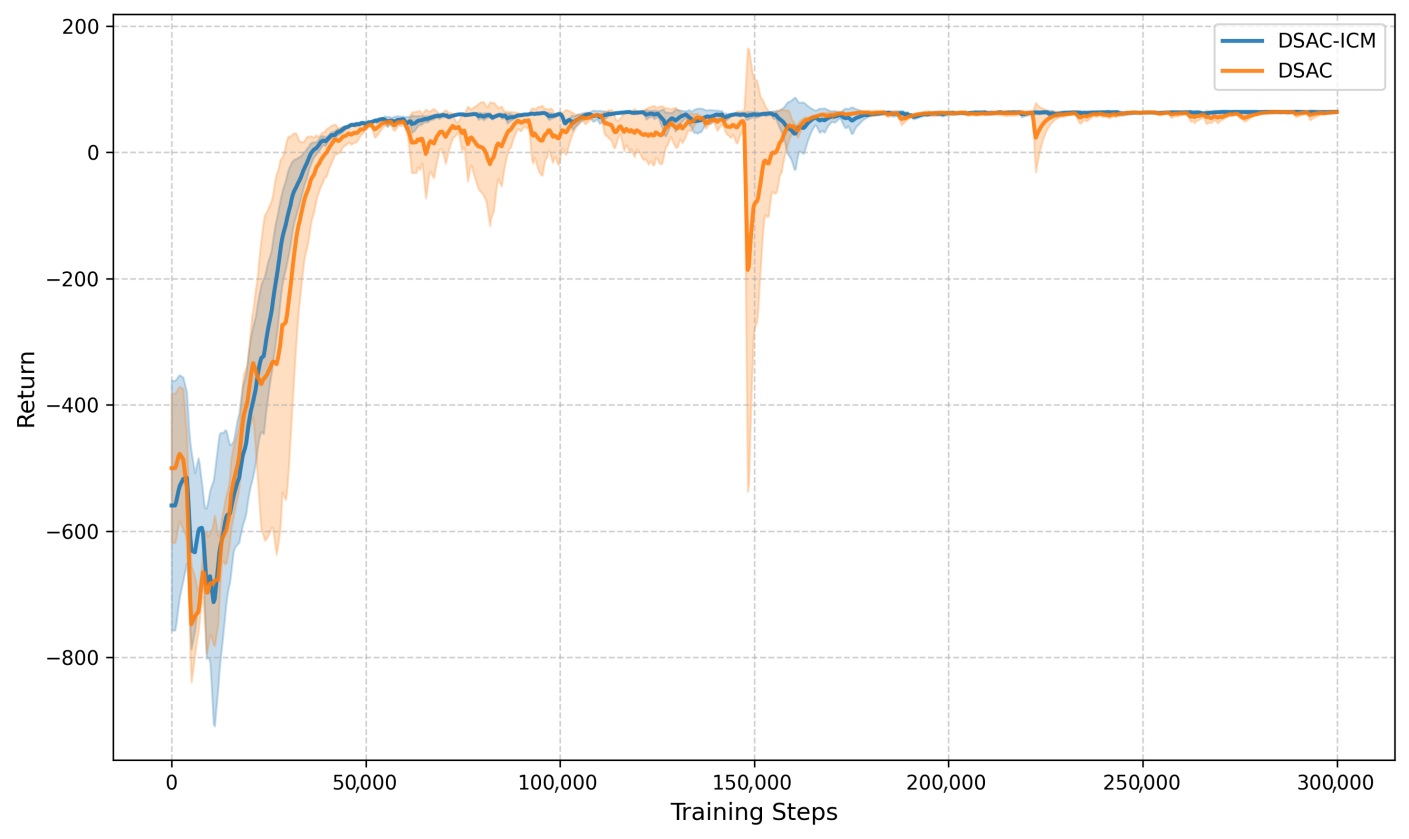
Training return curves of DSAC in the ICM ablation study. The horizontal axis denotes training steps, and the vertical axis represents episodic return; the blue curve corresponds to the framework *With ICM*, while the orange curve corresponds to *Without ICM*.

As shown in Figure 7, the two variants exhibit distinct learning dynamics. The *With ICM* variant (blue curve) shows larger fluctuations in the early exploration stage (even dropping to around −900), but it recovers quickly and converges to a stable high-return plateau (around 60) after approximately 50,000 steps. In contrast, the *Without ICM* variant (orange curve) exhibits milder fluctuations in the early stage, but suffers from frequent oscillations and occasional sharp drops in later training, particularly around 150,000 steps, indicating less robust exploration and policy optimization.

Table 4 further confirms the benefit of ICM in terms of final return: DSAC-ICM achieves a higher mean return (64.17) than DSAC without ICM (61.74). This contrast indicates that the ICM plays two core roles: it equips the agent to tolerate temporary exploration setbacks (reflected in the rapid recovery from deep early returns) and enhances the robustness of policy learning (reflected in the stable convergence of the *With ICM* variant). This confirms that ICM effectively promotes exploration in high-uncertainty terrain regions and contributes substantially to the overall policy quality.

Among the hyperparameters introduced by the Intrinsic Curiosity Module (ICM), the intrinsic reward weight λ plays a particularly important role, as it directly controls the balance between curiosity-driven exploration and task-oriented optimization. Compared to other hyperparameters, inappropriate choices of λ can more easily lead to either insufficient exploration or unstable learning dynamics. Therefore, we focus our sensitivity analysis on this parameter.

Specifically, we evaluate three representative values, λ∈{0.001,0.005,0.01}, while keeping all other hyperparameters unchanged.

**Figure 8:**
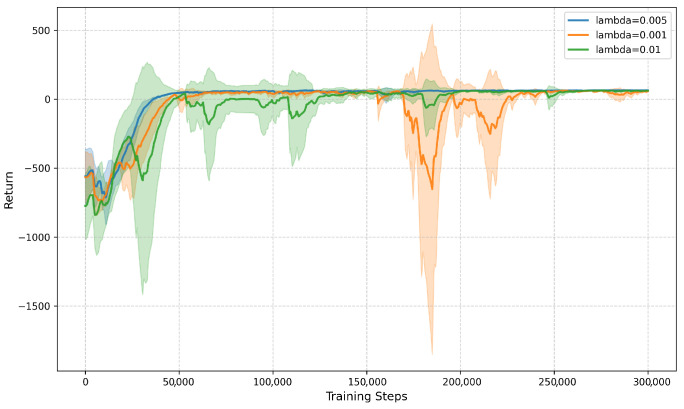
Training return curves of DSAC-ICM with different intrinsic reward weights λ. The horizontal axis denotes training steps, and the vertical axis represents episodic return; solid lines denote the mean return and shaded regions indicate one standard deviation across three runs.

As shown in Figure 8, when λ is too small (λ=0.001), the intrinsic reward provides insufficient exploration guidance, resulting in slower improvement and a lower final return. When λ is too large (λ=0.01), exploration becomes overly aggressive and introduces larger training fluctuations. In contrast, λ=0.005 achieves a better balance between exploration and exploitation, yielding more stable convergence and the highest final return.

Note that λ=0.005 is used as the default setting in all main experiments; therefore, its final return is consistent with Table 5.

## 6. Conclusions

We presented DSAC-ICM, a novel DRL framework designed to mitigate the inherent challenges of Q-value overestimation and inefficient exploration for path planning in 3D uneven terrains. By integrating distributional value learning with an Intrinsic Curiosity Module (ICM), DSAC-ICM achieves both algorithmic stability and exploration robustness.

Our experimental results lead to four main conclusions: Firstly, DSAC-ICM successfully enables the agent to learn robust strategies, generating high-quality, physically constrained paths in complex terrains. Secondly, compared to traditional planners like $A^{*}$, our method achieves the optimal trade-off between path quality and computational cost, making it suitable for real-time navigation. Thirdly, DSAC-ICM significantly outperforms all mainstream DRL baselines in convergence speed and asymptotic return. Finally, ablation studies confirmed that the ICM is crucial for enhancing exploration efficiency and accelerating convergence.

In summary, DSAC-ICM provides an efficient and stable solution for path planning in complex 3D environments. Future work will extend this framework toward more realistic navigation scenarios. Specifically, we plan to integrate DSAC-ICM as the global planner within a hierarchical planning architecture, where a local planner or reactive controller handles dynamic obstacles, perception uncertainty, and real-time disturbances. In addition, incorporating online terrain perception and validating the Sim-to-Real transferability on real robotic platforms will be important directions for future research.

## Figures and Tables

**Table 1:** Summary of key experimental hyperparameters for DSAC-ICM.

Parameter	Value
Discount factor γ	0.99
Target update rate τ	0.001
Actor learning rate	$3\times 10^{-4}$
Critic learning rate	$3\times 10^{-4}$
Entropy coefficient α	0.2
Batch size	256
Replay buffer size	20,000
Warm-up steps	2000
Training frequency	Every step
Gradient steps per update	1
Total training timesteps	300,000
ICM learning rate	$3\times 10^{-4}$
ICM feature dimension	64
ICM hidden dimension	64
ICM forward loss weight β	0.2
Intrinsic reward scale η	0.05
Intrinsic reward weight λ	0.005
Value network architecture	MLP [128, 128], GELU
Policy network architecture	MLP [128, 128], GELU
Action distribution	Tanh Gaussian

**Table 2:** Comparison of Path Planning Performance Across Methods.

Methods	PCR	PTR	CTT
Dijkstra	1.000	1.000	0.693
A*	1.000	0.196	0.145
RRT	1.159	0.870	0.606
3D-M	1.242	0.098	0.106
**DSAC-ICM (Ours)**	**1.137**	**0.056**	**0.055**

**Table 3:** Comparison of Training Performance Among Reinforcement Learning Algorithms.

Algorithm	Final Return (Mean ± Std)
SAC	59.91±2.69
M-RL	59.64±6.22
PPO	35.50±23.73
A2C	−1419.06±770.83
**DSAC-ICM (Ours)**	64.17±2.23

**Table 4:** Comparisons of final episodic returns in the DSAC ablation study (mean ± std).

Algorithm	Final Return (Mean ± Std)
DSAC	61.74 ± 0.82
**DSAC-ICM (Ours)**	**64.17** ± **2.23**

**Table 5:** Effect of intrinsic reward weight λ on final performance (mean ± std).

Setting	Final Return (Mean ± Std)
λ=0.001	58.68±6.47
λ=0.01	62.16±1.71
λ=0.005 **(Ours)**	**64.17** ± **2.23**

## Data Availability

The original contributions presented in this study are included in the article. Further inquiries can be directed to the corresponding author.
